# Comparison of median frequency between traditional and functional sensor placements during activity monitoring

**DOI:** 10.1016/j.measurement.2013.03.004

**Published:** 2013-08

**Authors:** Jeroen H.M. Bergmann, Selina Graham, Newton Howard, Alison McGregor

**Affiliations:** a Medical Engineering Solutions in Osteoarthritis Centre of Excellence, Imperial College London, London, United Kingdom; b Department of Surgery and Cancer, Imperial College London, London, United Kingdom; c Synthetic Intelligence Lab, Massachusetts Institute of Technology, Boston, MA, United States of America

**Keywords:** Accelerometry, Activity classification, Frequency domain, Placement, Signal processing

## Abstract

Long-term monitoring is of great clinical relevance. Accelerometers are often used to provide information about activities of daily living. The median frequency (*f*_m_) of acceleration has recently been suggested as a powerful parameter for activity recognition. However, compliance issues arise when people need to integrate activity recognition sensors into their daily lives. More functional placements should provide higher levels of conformity, but may also affect the quality and generalizability of the signals. How *f*_m_ changes as a result of a more functional sensor placement remains unclear. This study investigates the agreement in *f*_m_ for a sensor placed on the back with one in the pocket across a range of daily activities. The translational and gravitational accelerations are also computed to determine if the accelerometer should be fused with additional sensors to improve agreement. Twelve subjects were tested over four tasks and only the “vertical” *x*-axis showed a moderate agreement (Intraclass Correlation Coefficient of 0.54) after correction for outliers. Generalizability across traditional and functional sensor locations might therefore be limited. Differentiation of the signal into a translational and gravitational component decreased the level of agreement further, suggesting that combined information streams are more robust to changing locations then singular data streams. Integrating multiple sensor modalities to obtain specific components is unlikely to improve agreement across sensor locations. More research is needed to explore measurement signals of more user friendly sensor configurations that will lead to a greater clinical acceptance of body worn sensor systems.

## Highlights

• We compare two sensor placements (conventional and functional) for activity monitoring.
• Activity monitoring was measured by a powerful parameter (median frequency) that has recently been presented.
• The functional sensor placement showed agreement with the conventional one.
• We have shown that more functional placements of sensors can be used to measure relevant parameters.

## 1 Introduction

The challenges related to the ageing population are widely recognized. Although it may seem attractive to live longer, a good quality of life during those extra years is not guaranteed. Obtaining accurate information about the activities in everyday environments is therefore of great clinical relevance and could lead to further improvements in both preventative and rehabilitation approaches. There is currently a huge drive to develop mobile health systems, as a result of the many benefits associated with long-term monitoring of individuals in their home, and indeed community and work settings [1]. Mobile sensing devices provide the opportunity for clinicians and researchers to measure behaviour outside the laboratory and enhance ecological validity [2]. This is particular relevant for observing changes in activities of daily living (ADL), as these are an essential part of clinical frameworks. The International Classification of Functioning, Disability and Health (ICF) is the World Health Organization’s framework for measuring health and disability at both individual and population levels. Dysfunctions in ADL are considered to directly affect functioning and are seen as an important rehabilitation focus within the medical field.

However, monitoring devices used to measure ADL should not affect the normal daily behaviour of people, as this will influence their acceptance within the medical field [3]. Clinical technologies can only be sustainable if they adapt to users and interact with them in an intuitive manner. User preferences have often been overlooked at the early stages of device testing, especially in clinical research, despite the fact that they can fundamentally change the utility or design of the investigated system [4]. One possible option to improve patient compliance with novel measurement technologies is to integrate them with existing devices that have already been accepted and adopted by a wide range of people. Research has now slowly started moving into the realm of commercially available devices, such as smartphones. However, the motivation of applying smartphones in clinical research is still often related to the relative low-cost and high quality of the embedded electrical components. The arguably more important feature of allowing unobtrusive data collection is regularly neglected. Smartphones have the potential to measure performance continuously, without the need for changing normal daily behaviour [5]. The mobile phone has the added benefit that they are more discrete than a dedicated monitor device, which will reduce rejection due to the device’s poor aesthetic value and intrusiveness [6]. Recent evidence indicates that 48% of people in the USA that are aged above 75 years own a mobile phone [7], indicating the high level of acceptance already reached by these mobile devices.

Currently most smartphones contain one or more sensors. Several smartphones come with a built in tri-axial accelerometer, which can potentially be used to measure essential clinical parameters. Accelerometry has been used on a small scale to assess balance and attempts have also been made to investigate balance during functional tasks [8], [9], [10], [11]. Dedicated accelerometers are more frequently worn to monitor activities of daily living and can be used as a measurement of general health [12]. Many research groups still place wireless accelerometers approximately at the level of the centre of mass, located on the lower back at the S2 level of the Sacrum, as well as at the chest or thigh [13], [14], [15]. However, the placements of these accelerometers do not coincide with the location in which the mobile phone is normally kept. Information about how a more functional position would relate to a conventional placement is lacking. Determining how traditional and functional placements agree will help in generalizing outcomes and interpretations across sensor locations.

A literature review showed that very little information is available regarding the optimal sensor location for activity recognition or to what extent they agree (Appendix 1). One paper showed that sensor placement across four different locations indicated that the ankle yielded the highest activity recognition rate [16]. However, a study conducted with double the amount of subjects showed that the optimal placement for monitoring mobility was found to be on the upper leg [17]. Feature extraction and data mining algorithms were used in both these studies. A study that focused just on walking found that the most accurate location across a range of body types was placement of the accelerometer in the pocket [18]. They did compare “functional” placements of the belt, pocket and around the neck, but only focused on a step count parameter. Another study that looked at more gait parameters found that a head location provided a better outcome than placement on the legs [19]. None of these studies investigated an ecological placement of the sensor for activity recognition.

Different aspects of the acceleration signal can be explored, but this study will focus on the frequency domain, as the median frequency has recently been recognized as a promising method for activity recognition [20]. The ease at which median frequency can be calculated and the robustness of the analysis means this is a potentially important clinical parameter. The first aim of this explorative study is to determine how the median frequency of a traditional accelerometer placement (the back) compares with a more functional one (the front pocket). The hypothesis is that the direction of change in the median frequency of the accelerometer is independent of sensor placement. Subsequently, the question arises if the accelerometer should be the only sensor integrated into the system. There is an option to fuse together additional sensor modalities, such as gyroscopes and magnetometers [21]. An accelerometer will record translational and rotational inertial accelerations, as well as gravitational acceleration, as long as parts of these acceleration vectors are in line with the accelerometer’s axis of sensitivity [22]. The accelerometer will only provide the sum of these components making it hard to determine if the translational components should be obtained separate from the rotational components. A further partition between rotational and translation components can be performed by fusing several sensors together [21]. Gravitational, translational and the summed product of these two accelerations were computed using an optical tracking system to establish if separation between the components would increase agreement. It was hypothesized that the summed acceleration signal yielded an equal level of agreement, as the individual components.

## 2 Methods

### 2.1 Participants

Twelve healthy adults, seven men and five women, with a mean (range) age of 24 years (21–31) and a mean height and weight of 172 (152–185) cm and 70 (53–93) kg, voluntarily participated in this study. The protocol was approved by the College Research Ethics Committee and all subjects gave written informed consent previous to the experiment.

### 2.2 Activities

Participants were asked to stand still for 30 s, walk 4 m and climb a flight of stairs consisting of three steps. Only standing was timed, as the other tasks were measured for the duration it took to complete the activity. Subjects were asked to walk, ascend and descend the stairs at a self selected speed. The stair had a rise of 17 cm and a length of 20 cm from step to step. The width of each step was set at 60 cm. Each condition was measured three times per subject.

### 2.3 Apparatus

A wired triaxial accelerometer (Vernier Labpro, Oregon, US) was placed either on the back or the pocket during a range of functional tasks. Each of the sensitive axis of the accelerometer was calibrated beforehand using the rotational calibration method described by Krohn et al. [23]. However, instead of just orienting each axis to the earth’s gravity centre, several different orientations were explored and all measurements were repeated four times to obtain a more robust linear calibration equation.

**Fig. 1:**
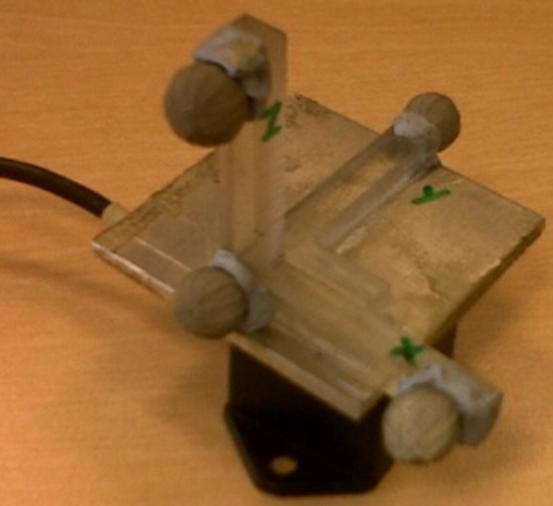
Marker cluster placed on the wired accelerometer. The markers were used for the construction of a local coordinate frame.

A passive optical tracking system (Vicon, Oxford, UK) was used to explore the potential changes in median frequency for each acceleration component separately. A custom-made coordinate frame consisting of four optical tracking markers was physically aligned with the accelerometer (Fig. 1). Each axis was initially represented by a 3D unit vector derived from a pair of markers.

Data were collected at a 100 Hz for both systems. The two devices were synchronized through a block pulse generated by the MX module of the Vicon.

**Fig. 2:**
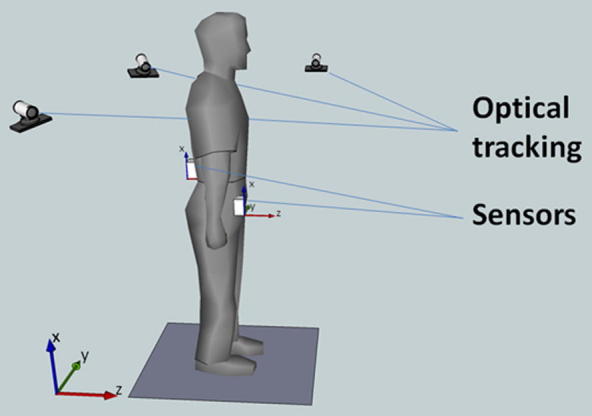
Experimental setup used including local (sensor based) and global coordinate frames.

The local coordinate frames were constructed in Matlab (MathWorks, Inc., Natick, MA, USA) during which the axes were redefined to align the coordinate system of the back with that of the pocket (Fig. 2). In short, the signs of the acceleration signal obtained from the *z* and *y*-axis on the accelerometer were inverted in order to align all local coordinate frames.

The marker cluster and sensor were placed directly on the back and on the outside of the pocket, as the passive markers needed to be visible to the cameras A stiff polymer case was placed in the pocket to mimic the smartphone. Displacement between case and sensor were checked before and after each trial.

Although, the marker frame was constructed with all arms perpendicular to one another, small alignment errors can be expected. To increase the accuracy of the representation of the local coordinate frame further computations were performed. Firstly, the dot product of each plane, that consisting of two vectors was calculated. The plane that yielded a dot product that was closest to zero was selected and the vector that was not part this plane was virtually re-established by calculating the cross product of the two remaining axes. The plane with the second lowest dot product outcome, which would include the previous computed axis, was identified and another new vector was calculated based on the two vectors that defined that plane. Finally, the two newly calculated vectors were used to determine the last vector by means of cross product computation. This method provided us with a coordinate frame that was truly perpendicular.

### 2.4 Gravitational acceleration

A vector was generated that represented the gravity vector. It started at the origin of the local coordinate frame, while running parallel to the vertical axis of the global reference frame. Subsequently, the amount of gravity measured by each of the sensitive axis was defined by the in plane angle between the gravity vector and each of the sensitive axes separately. A simple verification was performed by ensuring the summed accelerations of the axis produced a constant outcome of 9.81 m/s^2^.

### 2.5 Translational inertial acceleration

Translational accelerations were computed by a double differentiation of the origin of the local coordinate frame, within the global coordinate frame. Marker position data were low-pass filtered with a 4th order Butterworth [24] using a cut-off frequency of 10 Hz, before calculating the derivative. The same filtering was applied for the obtained velocity data before differentiation was performed. The amount of translational acceleration that ended up at each sensitive axis was established in utilizing the same method described for the gravitational acceleration. The rotational acceleration was not modelled as pilot data showed it to be very low for the range of tasks that were explored in this study.

### 2.6 Total acceleration

The gravitational and translational acceleration were added for each sensitive axis to obtain a total acceleration measure that could be compared to the values obtained by the accelerometer. A root mean square error (RMSE) between systems was calculated [2] for each trial.

### 2.7 Median frequency

The median frequency (*f*_m_) was calculated using a moving window method. The windows encompassed 3 s and they were shifted by one data point at each iteration, over the full length of the signal. A duration of 3 s was selected to allow this technique to be applied in future free living studies. It also covered a time period that fits patients whom pace might be lower than those of the healthy group presented in this study. All signals were offset against the mean of the first 50 data points, as subjects were requested not to move during this period. Apart from a short time interval (∼1 s) at the beginning and the end of the signal, the majority of the signal related to the task that was performed. Features in the frequency domain were investigated using the power spectral density [20] derived from the periodogram function in Matlab. The periodogram was chosen, since it is a computationally economic way of estimating the power spectrum. A one-sided (in frequency) power spectral density was calculated in units of power per radians per sample. The *f*_m_ was computed by firstly dividing the summed power of the windowed signal by two and subsequently determining the frequency at which the cumulative power exceeds the previous determined threshold value. The median value over all windows was obtained per trial, to ensure frequencies relating to the waiting element at the start and the end of each measurement did not affect the final result. The average value over all three trials was calculated and the concluding value obtained was then used for further analysis.

### 2.8 Statistical analysis

Agreement of *f*_m_ between the sensor locations was assessed using Intraclass Correlation Coefficients (ICCs) [25] and Bland and Altman analyses [26]. The ICCs were computed for the gravitational, translational and total acceleration. Bland and Altman plots were constructed to examine the difference between the two placements against the average value. The 95% limits of agreement were calculated and plotted using GraphPad Prism 5.0 (GraphPad Software, San Diego, California, USA). Indications of agreement, such as poor or moderate, were taken from [25].

## 3 Results

**Fig. 3:**
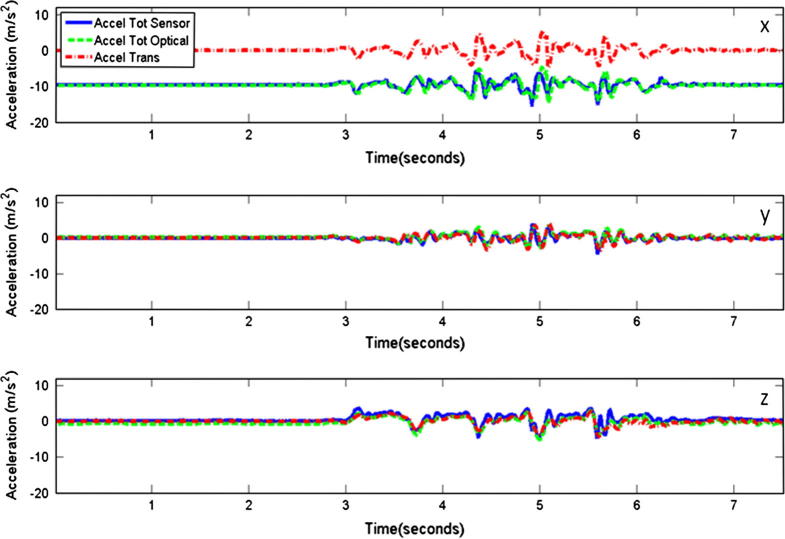
Example data illustrating the acceleration trajectories obtained from the two measurement systems. Data were collected at the pocket during a walking trial. The total accelerations obtained from the sensor (Accel Tot Sensor) and optical tracking systems (Accel Tot Optical), as well as computed translational accelerations (Accel Trans) are shown for each axis (*x*, *y* and *z*).

Accelerations between the two systems showed good correspondence (Fig. 3), as was expected based on results from other studies using similar techniques to determine accelerations from optical tracking data [24].

**Fig. 4:**
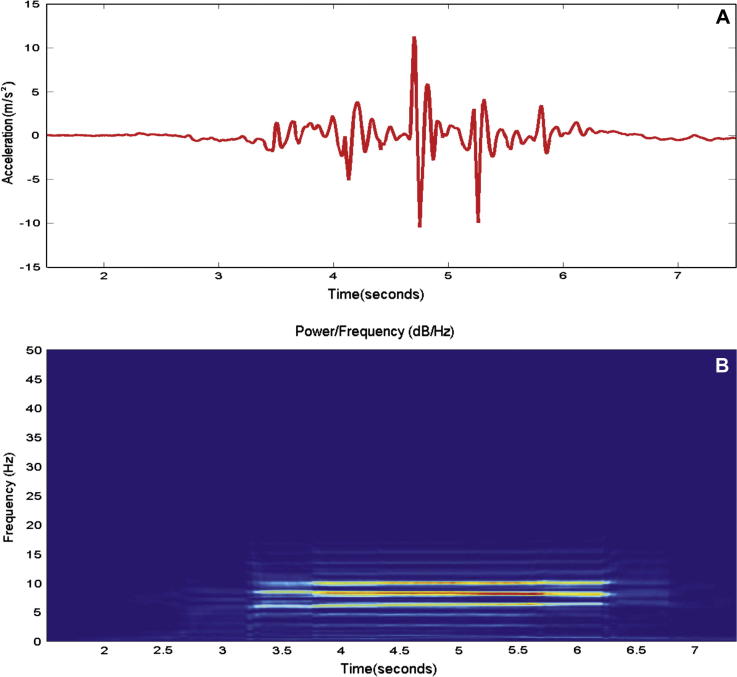
Graph A represents acceleration recorded from a sensor placed on the back. Data are shown for the *x*-axis only during a single walking trial. Graph B is the related power/frequency plot using a 3 second moving window.

An example of a walking trial and associated power/frequency using a moving window is given in Fig. 4. It shows the identification of the walking activity in the time–frequency plot devised using the previously described analysis method. It can be observed that the frequencies are rounded to the nearest discrete Fourier transform bin, which matches to the resolution of the signal. In this example the computed median frequency was 6.45 Hz over the whole duration.

The total acceleration had a moderate agreement between sensor placements for the *x*-axis (Table 1). The *y*- and *z*-axis had a fair and poor concurrence across the activities. An almost perfect agreement was found for the translational acceleration in *x* direction, but became fair after correction for outliers. The *y* and *z* directions only yielded a poor correlation for this component. The gravitational component yielded a poor relationship across all axes.

**Table 1:** The mean median frequency (± standard deviation) over all subjects given for each sensitive axis and activity.

Event	*f*_m_ (*x*-axis)			*f*_m_ (*y*-axis)			*f*_m_ (*z*-axis)		
	Accel tot (Hz)	Accel trans (Hz)	Accel grav (Hz)	Accel tot (Hz)	Accel trans (Hz)	Accel grav (Hz)	Accel tot (Hz)	Accel trans (Hz)	Accel grav (Hz)
Standing still	Back: 6.46 ± 2.58	Back: 7.35 ± 1.04	Back: 0.22 ± 0.18	Back: 4.05 ± 2.80	Back: 6.78 ± 0.80	Back: 0.34 ± 0.29	Back: 0.39 ± 0.61	Back: 6.44 ± 1.42	Back: 0.20 ± 0.15
	Pocket: 9.87 ± 4.03	Pocket: 7.16 ± 0.98	Pocket: 0.19 ± 0.26	Pocket: 3.70 ± 3.84	Pocket: 5.40 ± 1.74	Pocket: 0.21 ± 0.19	Pocket: 1.11 ± 3.20	Pocket: 5.55 ± 2.23	Pocket: 0.10 ± 0.10

Walking	Back: 4.79 ± 1.44	Back: 5.27 ± 1.19	Back:0.44 ± 0.33	Back: 5.43 ± 1.17	Back: 7.04 ± 2.78	Back: 0.47 ± 0.18	Back: 1.73 ± 0.48	Back: 1.86 ± 0.58	Back: 0.27 ± 0.15
	Pocket: 5.45 ± 1.30	Pocket: 6.36 ± 0.69	Pocket: 0.62 ± 0.21	Pocket: 4.04 ± 1.19	Pocket: 5.18 ± 1.23	Pocket: 0.41 ± 0.23	Pocket: 0.83 ± 0.38	Pocket: 3.47 ± 0.88	Pocket: 0.44 ± 0.14

Stair ascent	Back: 1.68 ± 0.79	Back: 2.13 ± 0.59	Back: 0.39 ± 0.14	Back: 0.97 ± 0.79	Back: 3.28 ± 1.29	Back: 0.38 ± 0.12	Back: 0.63 ± 0.27	Back: 2.33 ± 1.60	Back: 0.37 ± 0.15
	Pocket: 0.73 ± 0.44	Pocket:2.87 ± 0.84	Pocket: 0.27 ± 0.09	Pocket: 1.34 ± 1.35	Pocket:4.36 ± 1.46	Pocket: 0.44 ± 0.22	Pocket: 0.50 ± 0.54	Pocket:3.44 ± 1.49	Pocket: 0.25 ± 0.12

Stair descent	Back: 3.15 ± 1.38	Back: 3.26 ± 1.03	Back: 0.24 ± 0.08	Back: 3.54 ± 1.95	Back: 4.75 ± 1.43	Back: 0.39 ± 0.20	Back: 0.82 ± 0.40	Back: 2.52 ± 1.44	Back: 0.25 ± 0.13
	Pocket: 2.70 ± 1.35	Pocket: 3.37 ± 1.00	Pocket: 0.49 ± 0.26	Pocket: 2.78 ± 1.87	Pocket: 4.61 ± 1.28	Pocket: 0.36 ± 0.20	Pocket: 0.34 ± 0.14	Pocket: 4.69 ± 1.52	Pocket: 0.31 ± 0.10

ICC	0.5359	0.8106	0.164	0.3879	0.2334	0.0224	0.2948	0.2364	0.1681
ICC corrected	–	*0.266*	–	–	–	–	*0.0448*	–	–

The total acceleration (accel tot), translational (accel trans) and gravitational (accel grav) acceleration are given in separate columns for every axis. Intraclass Correlation Coefficients (ICCs) are given for each axis and acceleration component. *f*_m_ is median frequency. ICC corrected shows the values after correction for outliers identified by the Bland and Altman plots.

**Fig. 5:**
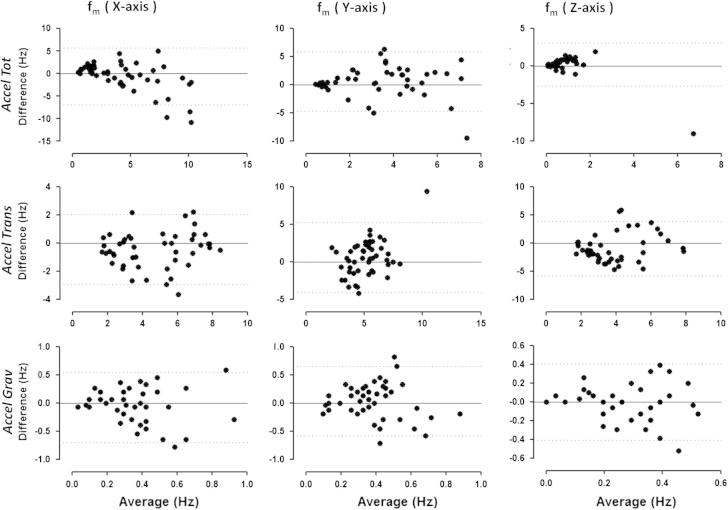
Bland and Altman plots given for the total acceleration (Accel Tot), translational (Accel Trans) and gravitational (Accel Grav) acceleration per sensitive axis.

The Bland and Altman plots (Fig. 5) showed that for the total acceleration output, the variation of the sensor location is dependent on the magnitude of the measurement. This was found across all axes. An outlier, as defined by [27], was identified for the *z* direction. Another outlier was observed for the *y*-axis of translational acceleration. No other systematic differences were observed across both the translational and gravitational accelerations.

## 4 Conclusions

Higher levels of agreement, between functional and conventional placements, will make it easier to compare outcomes across different assessment methods and patients. The aim of this study was to determine how the *f*_m_ of a traditional sensor placement compared to a more functional one. The ICCs value showed that after corrections for outliers, a moderate agreement was found for acceleration in the *x*-axis. Assessing these values on an ordinal scale showed that the median frequency between the two locations remained similar. This indicates that the direction that the median frequency shifts is independent of placement and strengthened the possibility of using more functional placements for activity monitoring or functional mobility tests.

Partitioning the signal into separate components diminished the overall agreement. This suggests that applying sensor fusion [21] to assess specific orientations and translations minimizes the generalizability of the obtained values across sensor locations. Applying multiple sensors will provide a richer dataset, but also allows for greater divergence between sensor locations. The overall recognition rate for activity monitoring is likely to increase by combining several sensors, but a fixed sensor placement might be needed to ensure this level accuracy. A single sensor system seems to provide a more robust method if locations are variable during activity monitoring. A single sensor device has the additional benefit that it will speed up data mining, decrease storage requirements and minimizes cost.

The *f*_m_ found by other researchers were similar to those obtained in this study. Chung et al. [20] reports a *f*_m_ during walking of 3.107 Hz (±0.534) for their research participant, which is similar to the findings for some of our participants, e.g. subject number 10 showed an *f*_m_ of 3.22 Hz (±1.03). Despite the positive association between these studies it still has to be noted that *f*_m_ differences between the optical tracking and accelerometer are probable. The optical tracking data has been filtered in order to obtain the acceleration signal, while the accelerometer signal has been kept original. This difference is likely to affect the *f*_m_ outcome, especially for the standing still task. Further deviations can be expected between the two systems, due to the motion artefacts of the optical tracking system that were not filtered out. Despite these limitations, the gathered data still classified well on an ordinal scale. Subsequently, the kind of accelerometer and processing techniques used will also affect outcomes, as it is known that the frequency responses depends on sensor type [28]. The accelerometer used in this study is a piezoelectric accelerometer with a similar frequency response and resolution as e.g. the LIS302DL MEMS iPhone accelerometer [29].

The placement of the sensor on top of the pocket might also provide slightly altered outcomes compared to a sensor placed in the pocket. However, displacement was checked and the displacements between the case placed inside the pocket and the sensor on top was very low. This was particular true for garments that fitted the leg more tightly. This suggests that the sensor closely mimicked the movements of the case (representing the phone) that was located in the pocket. The pocket placement was selected as a common location to place an everyday object. It has been shown previously that the pocket location has greater step count validity for a range of body types compared to placement on the belt or around the neck [18]. Another study that focused on activity detection found that the placement of a sensor on the upper leg yielded good results [17]. However, in that particular study the sensor was placed below the pocket and a strap was used to hold it in place.

The implementation of long-term monitoring techniques that utilize devices that are widely used and accepted are desirable from a user compliance point of view. Unobtrusive sensing has also been highlighted within the medical field as an essential feature to improve user acceptance of body sensor networks [3]. The mobile phone has the added benefit that it is more discrete than a dedicated monitor device, thus increasing user acceptance [6]. Patients may favour carrying the sensor device in their pocket, as it is less visible and more familiar. Smartphones have the potential to become a crucial tool for activity monitoring. Smartphones have been implemented to track a persons’ location, as well as to determine their level of activity [30]. In that particular study, physical activity was measured using an activity count method. This technique provides a more general overview of activity compared to the *f*_m_ based ADL detection. However, both methods could be combined to create a system with the ability to assess an individual in detail, as well as on a more generic level. It will even allow for GPS tracking to determine subsets of activities, such as driving.

This work demonstrated that more functional placements of sensors can yield acceptable agreement levels with traditional sensor locations. It suggests that everyday objects, such as smartphones, can be used to perform clinical relevant assessments. The study highlights the need of a more evidence-based approach for selecting sensor location, in which flexibility and ease of use are imperative.

## Appendix A Supplementary material

Supplementary data 1 Supplementary material contains Appendix 1.
