# Supplementary material for: Comparison of median frequency between traditional and functional sensor placements during activity monitoring
Source: Measurement (Lond). 2013 Aug;46(7):2193–200. doi: 10.1016/j.measurement.2013.03.004 (PMC4617466; doi:10.1016/j.measurement.2013.03.004)
Supplement: Supplementary data 1 — Supplementary material contains Appendix 1. [file mmc1.docx]

APPENDIX 1

Several previous studies, identified by a structured literature review, have looked at the effect of sensor locations on outcomes during measurements of activities of daily living (ADL). A literature search was performed consisting of a series of keywords that were established by the authors to identify studies and articles that might discuss different sensor placements for assessing ADL. The keywords, MESH headings and Boolean operators used for the electronic search are given here;

(Wearable sensor OR body worn sensors OR movement sensor OR motion sensor OR movement sensor OR inertia sensor OR wireless sensor OR accelerometer OR gyroscope OR Micro-Electrical-Mechanical Systems OR Actigraphy OR inertial measurement unit OR motion monitor OR body sensor network OR body area network) AND “Activities of Daily Living” **[**MeSH Term**]** AND (“placement” OR “location” OR “alignment”).

Using these keywords a search was initially conducted for relevant articles and conference proceedings using MEDLINE, EMBASE and Current Contents Connect, with articles being selected from 1966 and onwards. Results from each of these searches were initially screened according to their title. Those considered potentially relevant to the review were further assessed according to given abstract before full articles were obtained for those identified from this second round of screening. An additional search was performed to increase identification rate of papers, which discussed sensor locations for activity recognition, by cross referencing and reviewing relevant conference proceedings. A paper containing any outcome in any part of the manuscript that discussed the relevant topic was accepted

From the original 24 papers, four papers were identified as potentially useful after reviewing the title, abstract, and descriptors [[1-4](#_ENREF_1)]. The papers that were rejected focussed on falls, non-wearable sensors or assessed only one sensor location.

**References**

[1] A. F. Dalton*, et al.*, "A preliminary study of using wireless kinematic sensors to identify basic Activities of Daily Living," in *Engineering in Medicine and Biology Society, 2008. EMBS 2008. 30th Annual International Conference of the IEEE*, 2008, pp. 2079-2082.

[2] L. Atallah*, et al.*, "Sensor Positioning for Activity Recognition Using Wearable Accelerometers," *Biomedical Circuits and Systems, IEEE Transactions on,* vol. 5, pp. 320-329, 2011.

[3] N. A. SILCOTT*, et al.*, "Evaluation of the Omron HJ-720ITC Pedometer under Free-Living Conditions," *Medicine & Science in Sports & Exercise,* vol. 43, pp. 1791-1797 10.1249/MSS.0b013e318212888c, 2011.

[4] L. Atallah*, et al.*, "Detecting Walking Gait Impairment with an Ear-worn Sensor," in *Wearable and Implantable Body Sensor Networks, 2009. BSN 2009. Sixth International Workshop on*, 2009, pp. 175-180.
